# On-Line Monitoring of Biofilm Accumulation on Graphite-Polypropylene Electrode Material Using a Heat Transfer Sensor

**DOI:** 10.3390/bios12010018

**Published:** 2021-12-30

**Authors:** Andreas Netsch, Harald Horn, Michael Wagner

**Affiliations:** 1 DVGW Research Center, Water Chemistry and Water Technology, Engler-Bunte-Ring 9a, 76131 Karlsruhe, Germany; harald.horn@kit.edu; 2 Engler-Bunte Institute, Water Chemistry and Water Technology, Karlsruhe Institute of Technology (KIT), Engler-Bunte-Ring 9a, 76131 Karlsruhe, Germany; michael.wagner@kit.edu; 3 Institute for Biological Interfaces 1 (IBG-1), Institute for Biological Interfaces (IBG), Karlsruhe Institute of Technology (KIT), Hermann-von-Helmholtz-Platz 1, 76344 Eggenstein-Leopoldshafen, Germany

**Keywords:** heat transfer biofilm sensor, biofilm monitoring, bioelectrochemical systems, electrode materials

## Abstract

Biofilms growing on electrodes are the heart piece of bioelectrochemical systems (BES). Moreover, the biofilm morphology is key for the efficient performance of BES and must be monitored and controlled for a stable operation. For the industrial use of BES (i.e., microbial fuel cells for energy production), monitoring of the biofilm accumulation directly on the electrodes during operation is desirable. In this study a commercially available on-line heat transfer biofilm sensor is applied to a graphite-polypropylene (C-PP) pipe and compared to its standard version where the sensor is applied to a stainless-steel pipe. The aim was to investigate the transferability of the sensor to a carbonaceous material (C-PP), that are preferably used as electrode materials for bioelectrochemical systems, thereby enabling biofilm monitoring directly on the electrode surface. The sensor signal was correlated to the gravimetrically determined biofilm thickness in order to identify the sensitivity of the sensor for the detection and quantification of biofilm on both materials. Results confirmed the transferability of the sensor to the C-PP material, despite the sensor sensitivity being decreased by a factor of approx. 5 compared to the default biofilm sensor applied to a stainless-steel pipe.

## 1. Introduction

Biofilms are used in a variety of technical systems in a beneficial or productive manner, cleaning water in the wastewater treatment over a wide range from membrane bioreactors (MBRs) [1] to trickling filters [2]. Among these technologies in wastewater treatment are bio-electrochemical systems such as microbial fuel cells as new source of energy from wastewater [3] or microbial electrosynthesis cells for the production of base chemicals [4]. In microbial fuel cells, anodically grown electroactive biofilms oxidize organic compounds of the wastewater under anaerobic conditions, while in most studies in a separated cathodic chamber oxygen as electron acceptor is reduced [5,6]. The biofilms in microbial fuel cells create a protected environment for the electroactive bacteria, allowing for the bacteria to settle on the electrode surface, enabling the electron transfer between bacteria and electrode via conductive pili, cytochromes or electron shuttles [6]. The efficiency of electron transfer from the bacteria to the electrode is crucial for the overall performance of BES. Similar to other biofilm technologies the performance of microbial fuels cells depends on the morphological properties of the biofilm and an optimal biofilm structure (thickness, density) must be established to allow for a stable power output [7,8]. Bacteria with greater distance to the electrode do not further contribute to the current generation, as the electron transfer is constrained by the travel distance of the electrons from the bacteria to the electrode interface [7]. While increasing the biofilm density leads to an improved electrical performance, by reducing the electrical resistance of the biofilm and harboring more bacteria participating in the electron transfer the viability of biofilms is the highest near the anode. In a simulation, Kato Marcus et al. [9] showed that inert biomass such as dead cells or too thick biofilms on the anode deteriorate the electrical performance of a microbial fuel cell due to the substrate diffusion limitation to the surface of the electrode. Several groups of researchers have investigated the optimal biofilm thicknesses of MFCs. While an electroactive bacterium such as the *Geobacter sulfurreducens* in a monocultural biofilm can form thicknesses exceeding 100 µm [10], it was reported by Semenec and Franks [11] that in multispecies waste water biofilms bacteria located further than 50–70 µm from the electrode no longer contribute to the current production. Read et al. [7] reported a typical thickness for anodic biofilms in macro sized MFCs of approx. 50 µm.

Therefore, a biofilm sensor to monitor the accumulation of biofilm on the electrode is highly required for the optimization of biofilm control strategies in bioelectrochemical systems towards a stable power generation. Biofilm monitoring requires on-line, in-situ, non-invasive measuring methods that can be clearly attributed towards the formation of biofilms in the system [12]. Sensors for biofilm measurement, generally speaking, are based on a modified response of the accumulated biofilm to a signal of the sensor [13]. A series of different biofilm sensors have been presented in literature based on impedimetric [14,15], electrochemical [16,17], spectroscopic [18,19] or thermal methods [20,21]. Optical methods, such as confocal laser scanning microscopy (CLSM) [22,23] or optical coherence tomography (OCT) [24] for biofilm imaging, have been used for the quantification of biofilm growth. The application of these optical methods is mostly limited to lab-scale experiments as they need additional sample staining (CLSM) or are limited in their ability to be integrated as cost-efficient in-line sensors into industrial systems [25].

As each of these methods present different drawbacks or limitations such as detection range, the ability to distinguish between the compounds of the deposits, applicability and cost-efficiency for industrial systems, the choice of the sensor for the respective field of application must be evaluated in advance [12,13].

As cell attachment and biofilm development are dependent on the substratum [26], the biofilm thickness and structure are greatly influenced by the electrode material [11,27]. Hence, for a representative measurement of the biofilm the sensor should be directly applied on the electrode of a bioelectrochemical system. The characteristics of an electrode material have been extensively studied and require high conductivity, mechanical and chemical stability, large surface areas and good biocompatibility [28,29,30]. Carbonaceous based or metal-based materials have been suggested as the main types of anode materials. Due to its low costs, good electrical conductivity and large surface area, carbonaceous based electrodes have established themselves as a versatile most commonly used source of electrodes in microbial fuel cells [29,31]. Among the metal-based materials stainless-steel has distinguished itself as the most studied option due to its outstanding mechanical properties, corrosion resistance and low costs compared to noble metals [30]. Despite higher electrical conductivity than carbonaceous based materials, Dumas et al. reported lower maximum power densities for stainless steel electrodes used in microbial fuel cell [32]. Various groups reported, that plain stainless steel bioanodes inhibit bacterial adhesion due to their smooth surface and low surface area. With respect to microbial fuel cells (MFC) as energy producing BES such effects are unwanted as a robust electroactive biofilm is necessary to achieve high power densities [28,30,33]. Other biofilm sensors being also applied in industry are based on electrochemical methods (ALVIM sensor [16]) or impedance spectroscopy [14]. These require an interface made of a material different than the material of an electrode used in BES. For the ALVIM sensor a stainless-steel electrode was used [16], while for the sensor reported by Pires et al. [14] a gold electrode served as substratum. Thereby, the different properties of the substratum may diminish the immediacy of the sensor output. Another industrially applied biofilm sensor is integrated into the OnGuard 3B Analyzer from Solenis (www.solenis.com/de-de/research-and-development/innovationen/onguard-3b-analyzer-for-biofouling (accessed on 09 December 2021)). This sensor combines ultrasound biofilm thickness measurement with the detection of deposits based on the reduction of heat transfer. However, this sensor is installed in a side stream and does not allow adapting to electrode materials.

Monitoring of biofilm accumulation in BES requires a rather simple, robust and reliable detection mechanism as well as the development of biofilm on materials suitable for the application as electrodes. Additionally, sensors must not alter flow conditions in order to analyze representative biofilm structures. Biofilm sensors based on impedimetric [14] or spectrometric methods [18] are often limited to laboratory conditions, whereas control of BES operation is required at technical scale.

In the current study thus a heat transfer biofilm sensor (DEPOSENS from LAGOTEC GmbH (Magdeburg, Germany)) was applied for the monitoring of biofilm accumulation on an electrode material. Briefly, results presented here contribute to a research project where electrodes made from C-PP will be used in MFC and equipped with DEPOSENS biofilm sensors to correlate biofilm development and MFC performance.

DEPOSENS biofilm sensors were tested in parallel in a stainless-steel pipe and a pipe constructed out of composite graphite-polypropylene (C-PP) to estimate this sensor for the use in BES applications [34].

## 2. Materials and Methods

### 2.1. DEPOSENS Biofilm Sensor

**Figure 1:**
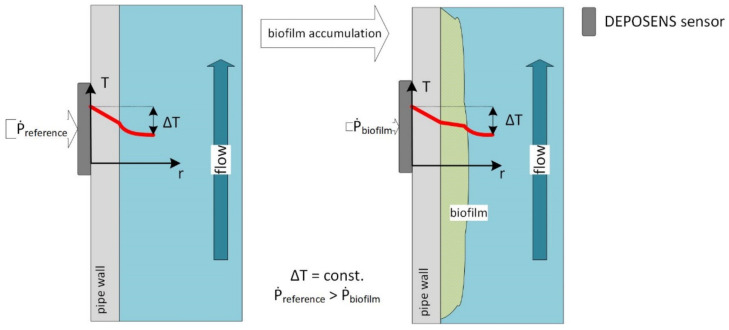
Measurement principle of the DEPOSENS biofilm sensor: the sensor board, consisting of one heater and two temperature probes, is glued to the outside of a pipe wall with a thermally conductive adhesive. Accumulating deposits (e.g., biofilm) on the pipe wall increase the thermal resistance. Consequently, the sensor requires a smaller heating power $\dot{P}$ to maintain the constant temperature difference ∆T.

Experiments were conducted using DEPOSENS biofilm sensors manufactured by Lagotec GmbH (Magdeburg, Germany). The measurement principle of the sensor is displayed in Figure 1. It is based on the increase of the thermal resistance R_th_ of a pipe caused by deposits (e.g., biofilm) accumulating on the inner wall of the pipe (1). Deposits such as calcium carbonate ($\lambda_{{\mathrm{CaCO}}_{3}}$ = 2.6 W∙m^−1^∙K^−1^) or biofilms (λ_biofilm_ = 0.6 W∙m^−1^∙K^−1^) [35] have much lower thermal conductivities in relation to the thermal conductivity of the stainless-steel pipe wall (λ_SST_ = 13.31 W∙m^−1^∙K^−1^). Therefore, the accumulation of such deposits impairs the heat transfer through the pipe wall and correlates proportionally to the thickness of the deposited layer. This phenomenon is commonly observed in heat exchangers and accounts a loss of heat transfer efficiency [36].

(1) $$R_{th}=\left(\frac{ln\left(r_{outer}\right)-ln\left(r_{inner}\right)}{\lambda_{pipe}}+\frac{ln\left(r_{inner}\right)-ln\left(r_{biofilm}\right)}{\lambda_{biofilm}}\right)\cdot \frac{1}{2\pi l}$$

The sensor (in Figure 1) consists of a heater and two temperature probes at different longitudinal positions. These components contact the outer wall of the pipe through a thermally conductive adhesive. The sensor is surrounded by a thermally isolating PUR-cover. One probe is measuring the pipe wall temperature near the heater while the other measures the temperature of the medium through the pipe wall. The sensor is not reaching into the pipe. Therefore, the biofilm development in the pipe is not manipulated by locally altered hydrodynamic conditions. A fixed temperature difference between heater and medium is set, thus a heat transfer from the heater to the medium is initiated. Proportionally to the accumulated deposits, the thermal conductivity from the heater through the pipe wall to the medium is reduced. Composition, thickness and density impact the thermal conductivity of the deposit layer. Consequently, this decreases the necessary amount of heating power to establish the set temperature difference ∆T between the two temperature probes. The resulting signal is given in reference to an initially deposit-free pipe. The flow rate must be maintained constant, as a change in the flow rate would increase or decrease the amount of heat extracted by the water, thereby altering the sensor measurement.

### 2.2. Experimental Setup and Biofilm Cultivation

Within this study temperature differential settings of ∆T = 10 K, 5 K and 2 K were evaluated. An overview of all performed experiments with the respective parameters is listed in Table 1.

As previously mentioned, two different pipe materials were tested: the commercially available 1.4571 stainless-steel pipe (SST) and an electrically conductive graphite-polypropylene-compound pipe (C-PP) (80% graphite, 20% polypropylene). This material was also used by Muddemann et al. [34] as an electrode material for bioelectrochemical systems. The SST pipes had a length of 250 mm, whereas the C-PP pipes were 300 mm long with inner diameters of 25.4 mm. Since the thermal conductivity of the C-PP material (λ_C-PP_ ≈ 21 W∙m^−1^∙K^−1^) and that of the SST material (λ_SST_ = 13.31 W∙m^−1^∙K^−1^) [37] are in a similar magnitude, a good transferability of the biofilm sensor from the standard SST material to the C-PP material was expected. However, the increased thermal conductivity of the C-PP material may impact the quality of the sensor’s measurement.

**Figure 2:**
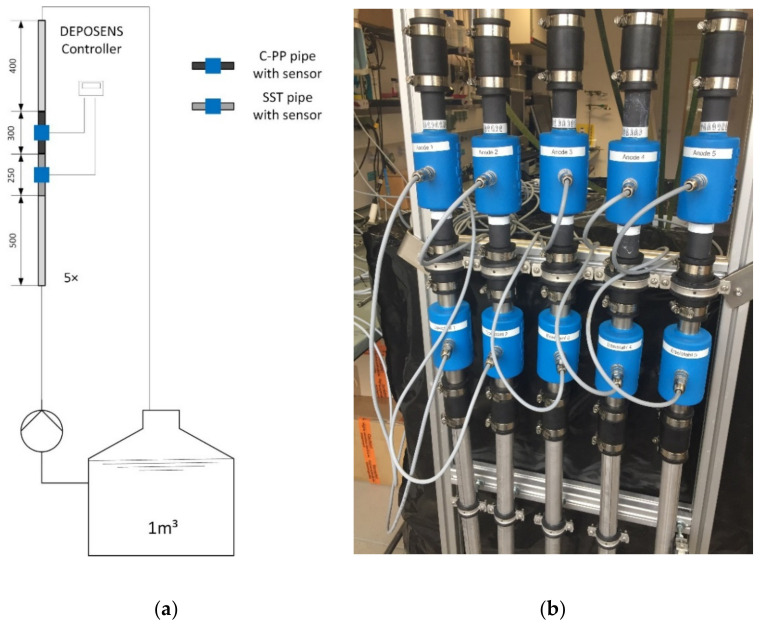
(**a**) The flow diagram of the experimental setup is shown. (**b**) A photograph of the lab-scale setup with five parallelly operated pipes including each one SST pipe (bottom) and one C-PP pipe (top) with biofilm sensors installed in series.

The DEPOSENS biofilm sensors were installed into a recirculatory piping system with five pipes operated in parallel, containing each one SST pipe and one C-PP pipe in series. The experimental setup is shown in Figure 2 Each pipe (inner diameter 25.4 mm) was equipped with a magnetic gear pump (Niemzik PAT, Haan, Germany) recirculating the cultivation medium. Biofilm cultivation was conducted under turbulent hydrodynamic conditions (Re = 3000; u = 12 cm/s). To ensure fully developed turbulent hydraulic conditions at the point of measurement of both sensors a run-in distance of 500 mm and a run-off distance of 400 mm was installed.

After a 24 h inoculation phase with 120 L of activated sludge supernatant from the nearby wastewater treatment plant Bruchsal (Germany), the cultivation medium (V = 1000 L) was added to the recirculatory system. During the first 48 h of the experiments, the average flow velocity was set to u = 6 cm/s (Re = 1500) to improve bacterial adhesion in the early stage of biofilm formation, due to the reduced shear stress [38]. Afterwards, the average flow velocity was increased to u = 12 cm/s (Re = 3000).

For the cultivation medium, a molar C:N:P-ratio was chosen at 100:10:1 as an optimal nutrient supply for biofilms growing under anaerobic conditions, as they can be found in the anodic chamber of microbial fuel cells. The cultivation medium (based on tap water) had an initial chemical oxygen demand (COD) of 200 mg/L and N$H_{4}^{+}$-N of 8.24 mg/L. Sodium acetate was chosen as carbon source (c = 238.5 mg/L) and ammonium chloride (c = 31.1 mg/L) as nitrogen source. A K_2_HPO_4_/KH_2_PO_4_ mixture was used to buffer the cultivation medium at pH = 7.5. COD, ammonium and phosphate concentration were measured every 48 h with Hach Lange vial tests, as well as the pH value and dissolved oxygen concentration. Substrate and nutrients were added to the cultivation medium when COD fell below 20 mg/l or N$H_{4}^{+}$-N concentration was less than 1 mg/L.

### 2.3. Gravimetric Biofilm Characterization

Following the cultivation of the biofilms in the pipes for up to 26 days until the DEPOSENS biofilm sensor signal did no longer indicate further biofilm accumulation, the pipes were sampled to determine the mean biofilm thickness and biofilm density. The pipes were drained for 10 min in vertical position before weighing the pipes in order to determine the wet mass. Afterwards, the wet biofilm was scrapped off the pipe for the determination of biofilm wet density as well as organic and inorganic fractions. When detachment of the deposits (biofilm) was visible during draining the particular pipe was withdrawn. This was the case for two pairs of pipes at 10 K applied temperature difference and for one pipe pair at 5 K temperature difference.

The mean biofilm thickness was calculated, according to Equation (2) with *m* corresponding to the mass of the pipe in a clean state and with biofilm, respectively; *A* corresponds to the inner surface of the pipe.

(2) $${\bar{L}}_{F,grav}\frac{m_{pipe\;with\;biofilm}-m_{clean\;pipe}}{A_{pipe}}\times \rho_{water}$$

The mean biofilm density ${\bar{\rho }}_{F,grav}$ was calculated according to Equation (3):

(3) $${\bar{\rho }}_{F,grav\;}=\frac{m_{F,dry}}{m_{F,wet}}$$

The fraction of inorganic compounds $\varepsilon_{inorg}$ was determined according to Equation (4):

(4) $$\varepsilon_{inorg}=\frac{m_{F,dry}-m_{F,ash}}{m_{F,wet}}$$

### 2.4. Data Analysis and Quality Control

The aim of this study was to correlate the sensor signal with the morphology of the accumulated biofilm in the C-PP and SST pipes, respectively. After termination of the experiments the mean biofilm thickness was correlated with the mean sensor signal of the final hour of the experiment (12 measurements). The data were plotted and a linear fit was applied to determine the sensitivity of the sensor. The linear fit was forced through the origin, because no biofilm was present at the time of sensor calibration. Resulting from the slope of the linear fit the sensitivity $\cdot {\bar{L}}_{F,grav}/\cdot sensor\;signal$ was determined.

Due to the small sample size Shapiro–Wilk and Kolmogorov–Smirnov test were performed determining the distribution of the variables mean biofilm thickness, mean biofilm density and fraction of inorganic compounds for both the ∆T = 10 K and ∆T = 5 K settings. Followed by a Grubbs test to identify outliers in the data. More details are shown in Tables S2 and S3.

## 3. Results

**Figure 3:**
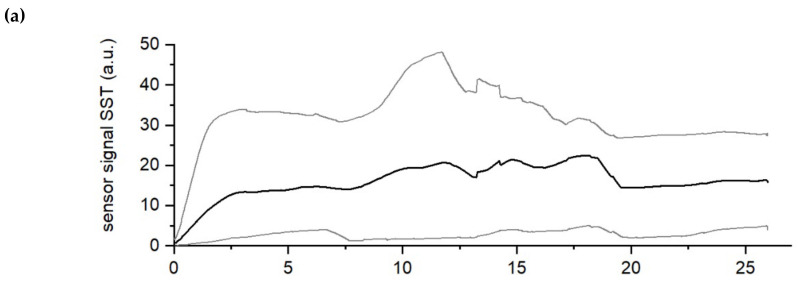

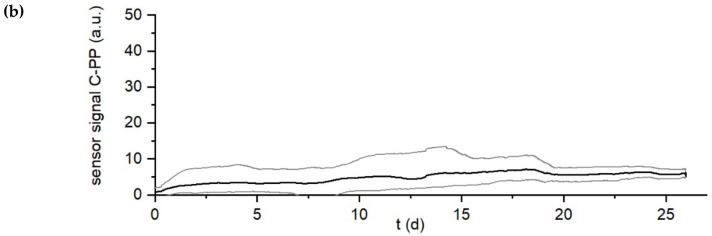
Development of the DEPOSENS sensor signal on the pipe made of SST (**a**) and the C-PP pipe (**b**) for all experiments (*n* = 9) at standard conditions (∆T = 10 K). Due to the short measurement interval (5 min) a moving average was applied over a timespan of one day. Day 0 marks the end of inoculation at which the flow velocity was increased to 12 cm/s (Re = 3000). The thick black line displays the mean signal (in auxiliary units (a.u.)) for all experiments, while the light grey lines display the maximum or minimum signal from any of the sensors at the respective time.

Biofilm accumulation was monitored for 26 days until the DEPOSENS signal reached steady state. As biofilm accumulated the heat transfer resistance from the heater to the medium increased, resulting in the recorded signal. Figure 3 shows the minimum, maximum and mean signal output of both sensors for all experiments (*n* = 9) at the standard temperature differential setting of ∆T = 10 K (see Table 1).

Due to the high measurement interval (5 min) a moving average of the sensor signal over a timespan of one day was applied. In Figure 3 it can be seen that while the course of the sensor signal for both sensors is comparable, there is a difference in the intensity of the measured signal. Over the course of the experiment for both the biofilm sensor on the SST and the C-PP pipes the sensor signals increased steadily and reached a plateau after approx. day 15. As the flow velocity was constant the signal was clearly attributed to the accumulation of deposits (e.g., biofilm) inside the pipes. However, the mean value of the signal (black curves in the Figure 3a,b respectively) of the biofilm sensor on the SST pipe increased more rapidly and steeper to approx. 20 a.u. while the mean signal of the biofilm sensor on the C-PP pipe grew more steadily to approx. 6 a.u. for all the conducted experiments (*n* = 9). It can be concluded that the biofilm sensor on the C-PP pipe is able to display a growth curve of the sensor signal, which follows a comparable trend to that of the sensor signal from the sensor applied to the SST pipe, despite being of smaller magnitude. Nevertheless, these findings indicate that the C-PP material is applicable to the sensor for the monitoring of a biofilm accumulation, in settings where stainless steel as material cannot be applied (e.g., on electrodes of BES).

For the purpose of sensor application, the sensitivity (biofilm accumulated per sensor signal a.u.) must be determined, to be able to translate the sensor signal value (measured in auxiliary unit (a.u.)) into the mean accumulated biofilm thickness $\bar{L}$_F_ (µm). Assuming an equally distributed biofilm accumulation over the total area of the respective pipes with integrated sensor the gravimetrically determined mean biofilm thickness represents the mean biofilm thickness at the point of the sensor measurement. Since the sensor signal is based on the heat transfer through the biofilm, not only the thickness of the accumulated biofilm but also the biofilm density or fraction of organic and inorganic compounds affect the heat transfer, therefore the sensor signal. In Table 2 the gravimetrically determined characteristics of the biofilms in both pipe materials are summarized.

A total of nine pairs of pipes with sensor were investigated throughout the experiments at the standard temperature difference ∆T = 10 K. At the end of the experiments large variations of the gravimetrically determined biofilm characteristics (mean thickness, mean wet density and inorganic fraction) was observed among the individual pipes. Yet the mean biofilm densities and inorganic fractions for both the C-PP pipes and SST pipes were similar. The mean biofilm thickness $\bar{L}$_F_ on the other hand indicates the tendency to accumulate thicker biofilms in the C-PP pipe. The mean biofilm thickness in the C-PP pipe exceeded the biofilm thickness in the SST pipe by 63%.

**Figure 4:**
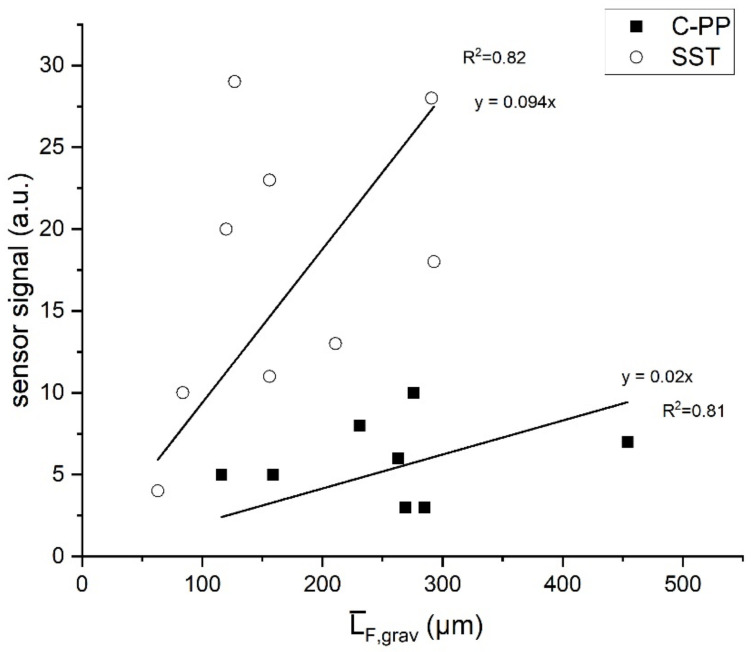
Correlation of the mean sensor signal of the final hour of the experiment (12 measurements) with the gravimetrically determined mean biofilm thickness accumulated in the SST and C-PP pipes with integrated biofilm sensors (∆T = 10 K). Assuming that no biofilm was accumulated in the pipe at the time of sensor calibration the linear correlation was forced through the origin.

Despite the sensor signal of the biofilm sensor applied to the SST pipe to exceed the signal of the biofilm sensor on the C-PP pipe by 2-3-fold (Figure 3), on trend less biofilm has accumulated in the SST pipes indicating different sensitivity of the sensors depending on the material of the pipe. For the determination of the sensor sensitivity the mean gravimetrically determined biofilm was correlated to the mean signal of the final hour (12 measurements) of the respective sensor. This correlation is plotted in Figure 4. Before each experiment the pipes were cleaned and a new reference for the sensor signal was set at 0. The linear fit function was forced through the origin, since at the start of the experiments with sensor signal 0 no biofilm was accumulated in the pipes. For the standard temperature difference (∆T = 10 K), the coefficients of determination were R^2^ = 0.82 and R^2^ = 0.81, respectively. The sensitivity of the sensors was 11 µm/a.u. (on the SST pipe) and 50 µm/a.u. (on the C-PP pipe), respectively, showing that the sensitivity of the sensors applied to SST pipes exceed the sensitivity of the sensors on C-PP pipes by almost a factor of 5.

### Influence of Setting of Temperature Difference

The experiments were repeated at different temperature differences ∆T of 2 K and 5 K, respectively, addressing the effect of ∆T on the sensor signal. By applying lower temperature differences, the aim was to reduce the effect of longitudinal heat transfer along the pipe wall, which potentially interferes with the temperature measurement of the medium temperature sensor (see Figure 1). An increased longitudinal heat transfer in the C-PP pipes compared to the SST pipe was expected due to the higher thermal conductivity of the C-PP material. A reduction of the longitudinal heat transfer could improve the sensitivity of the sensors.

For the 2 K setting the biofilm accumulation was performed in a total of four sensor pairs. Biofilm accumulated well in both the SST and C-PP pipes with a mean biofilm thickness of 161 ± 52 µm and 302 ± 59 µm. These results are comparable to those obtained from the pipes with the 10 K setting of the sensors. As a consequence of the lower temperature difference between heater and medium the sensitivity of sensors on both materials decreased, resulting in sensor readings in the range of 0 to 5 a.u. Within these narrow ranges of the sensors output signals at a 2 K setting, the biofilm accumulation in the pipe cannot be displayed well by the sensor anymore. The 2 K setting of the temperature difference was therefore not further investigated.

**Figure 5:**
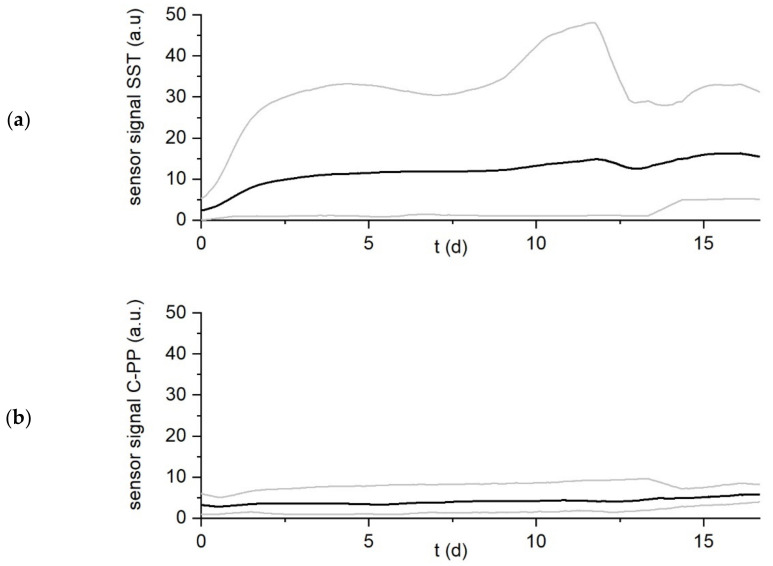
Development of the DEPOSENS sensor signal on the pipe made of SST (**a**) and the C-PP pipe (**b**) for all experiments (n = 9) at standard conditions (∆T = 5 K). Day 0 marks the end of inoculation at which the flow velocity was increased to 12 cm/s. The thick black line displays the mean signal for all experiments, while the light grey lines display the maximum or minimum signal from any of the sensors at the respective time.

With the applied temperature difference of 5 K a total of eight pairs of pipes with biofilm sensors were investigated, showing a similar response of the sensor in terms of readings and sensor sensitivity compared to the 10 K setting. The development of the sensor signal is shown in Figure 5. The resulting sensitivities of the biofilm sensors for all applied temperature differences are summarized in Table 3 for the SST pipes and C-PP pipes, respectively. Thereby, showing that the sensitivity did not improve by reducing the applied temperature difference to ∆T = 5 K.

## 4. Discussion

As previously mentioned in reviews by Janknecht and Melo [13] or Flemming [12] biofilm monitoring devices requires to feature an on-line non-invasive mechanism to display the accumulation of biofilms within a technical system. The aim of this study was to investigate the application of the heat-transfer DEPOSENS biofilm sensor on a carbonaceous-based electrode material (C-PP) for BES. The application on the C-PP material gives the major advantage of eliminating the influence of a different substratum to the growth of the biofilm, compared to the application of DEPOSENS sensor to stainless steel (SST). Due to its drawbacks stainless steel is not commonly utilized as electrode material in microbial fuel cells [28,30]. Furthermore, with the application of the sensor to the C-PP material the sensor has the potential to be installed directly on the electrode. Thereby, the sensor is able to monitor the biofilm growing at the hydrodynamic pattern at the surface of the electrode. Usually, heat-transfer biofilm sensors have been installed to a side-stream in an industrial plant [13]. Thereby, limiting the accuracy as the hydrodynamic pattern in the side stream may not be identical to the flow conditions inside the pipe or reactor. As reported by Recupido et al. [39], the morphology of a biofilm is influenced by the hydrodynamic conditions, thus an installation of the sensor into a side stream of the plant may decreases the ability of the sensor signal to represent to actual situation on the electrode.

Heat-transfer biofilm sensor, like the DEPOSENS sensor, require constant flow conditions, since changing flow velocities of the medium impact the amount of heat withdrawn from the system. For example, a sudden increase of the flow rate results in a steep drop of the sensor signal, as the heater of the sensor requires more power to maintain the temperature difference ∆T. A higher power input to the heater corresponds to a thinner deposit layer and vice versa. Otherwise, a correction factor is necessary to compensate for the different flow velocities. In the targeted application of the sensor on the fixed anode in a microbial fuel cell, constructed as a rotating disk reactor with rotating cathode [40], at the position of the sensor the flow velocity is constant, thereby diminishing the need for a correction factor.

Two of the major drawbacks generally reported by Janknecht und Melo [13] are the low sensitivity of heat transfer sensors due to high uncertainties of the measurement of the wall temperature and the inability to distinguish between the compounds of the deposits, can be seen by the results reported in this study. Garcia et al. [36] reported in their research on the impact of biofilms on the heat transfer in seawater tubular heat exchangers that although the majority of the biofilm is composed of water (0.6 W∙m^−1^∙K^−1^), its thermal conductivity may be increased by the concentration and nature of the solid composition of the biofilm. A decrease of the thermal conductivity was observed with a reduction of the solids present in the biofilm from 4.7 mg∙cm^−2^ to 2.2 mg∙cm^−2^. In comparison to the accumulated biofilms in this study the biofilms (0.32 mg∙cm^−2^) in the C-PP pipe and 0.36 mg∙cm^−2^ in the SST pipe at 10 K temperature difference) investigated by Garcia et al. [36] showed a much higher concentration of dissolved solids in the biofilm. Otherwise, Characklis et al. [41] reported no significant correlation between the heat transfer coefficient of a biofilm and its density, while investigating biofilms with similar properties to the accumulated biofilms in this study. In this study a linear correlation between the biofilm thickness and the sensor signal was assumed, which is in agreement with the reports of Janknecht und Melo [13] stating that a biofilm thickness of 10 µm will increase the overall thermal resistance by 1–1.5%. The effects of biofilm density and inorganic fraction on the sensor’s sensitivity could not be quantified.

With the application of the sensor on the C-PP material, a reduction of the sensor’s sensitivity by approx. 80% in comparison to that on the SST material was observed. This observation can largely be explained with the higher thermal conductivity of the C-PP material (21 W∙m^−1^∙K^−1^ vs. 13.3 W∙m^−1^∙K^−1^). As previously explained two temperature probes are located in the sensor board on different longitudinal positions, measuring the temperature of the heater and the medium temperature, respectively. The heat flow from the heater is not limited to across the pipe wall, but it will also transfer heat longitudinally along the pipe wall to the medium temperature sensor. Due to the increased thermal conductivity of the C-PP material this proportion of heat interferes with the temperature measurement to a greater scale than on the SST material. Thus, the sensitivity of the sensor on the C-PP material is diminished. To reduce the effect of the longitudinal heat transfer, a smaller temperature difference would be preferential, but as shown in Table 3, the sensitivity of the sensor did not improve on either the C-PP or SST material with a lower temperature difference.

In microbial fuel cells an optimal biofilm thickness must be established to allow for efficient electron transfer and substrate access [8], since only live cells can contribute to the current generation. Ge and He [42] have investigated the long-term performance of MFCs with wastewater and reported an unstable and deteriorating performance. To stabilize the MFC performance Islam and coworkers have therefore applied two different biofilm control mechanisms in microbial fuels cells with ultrasound [43] or with flushing [44] in order to control the biofilm thicknesses accumulated on the electrodes. The herein described sensor can be used as a trigger for the application biofilm control mechanisms. Several groups of researchers have investigated the biofilm thicknesses in MFCs. For the biofilm sensor to be an effective monitoring tool to trigger flushing procedures, the sensor must be able to identify the threshold of excessive biofilm thickness, which would alter the performance of a BES. Given the reported optimal biofilm thicknesses for microbial fuel cells of approx. 50 µm [7,11], the low sensitivity of the sensor, when applied to C-PP, may limit its ability to display the precise biofilm thickness on the electrode. An improvement of the sensitivity of the sensor on C-PP would be desirable.

## 5. Conclusions

The aim of this study was to investigate the applicability of the DEPOSENS biofilm sensor on a graphite-polypropylene (C-PP) material in comparison to the standard stainless-steel (SST) pipe application, as a direct monitoring device of biofilms developing on the electrode of BES made from the same conductive composite material. This work has shown the following.

- The DEPOSENS biofilm sensor is able to identify an accumulation of biofilm on the inside of the pipe on both stainless steel and C-PP corresponding to the thickness of the accumulated biofilm. The application of the sensor on C-PP is needed for electrodes made from C-PP to have comparable biofilm growth characteristics in pipe sensors and on electrodes in BES.
- The application on the C-PP material rather than the standard stainless-steel pipe resulted in a reduction of sensitivity of the sensor, despite fairly similar thermal characteristics of the materials. The sensors on the C-PP material displayed a sensitivity (50 µm/a.u.) approximately 5-fold less than the sensor on stainless-steel (11 µm/a.u.)
- The reduced sensitivity limits the application of sensor on C-PP to technical systems with accumulating biofilm thicknesses of greater than 50 µm.
- The recommended operational settings for the application of the sensors with a temperature difference of minimum of 5 K.

## Figures and Tables

**Table 1:** Overview of experimental conditions (stain steel pipe, SST and graphite-polypropylene-compound pipe, C-PP) with a pipe diameter of d_i_ = 25.4 mm, cultivation time up to 26 days. In total, five different runs were performed. A list with the different runs is provided in the Table S1 in Supplementary Materials.

Q (L/min)	u (cm/s)	Re (–)	∆T (K)	Number of Replicates Used
3.6	12	3000	10	9
			5	8
			2	4

**Table 2:** Comparison of the gravimetrically determined mean biofilm thickness, biofilm density and fraction of inorganic compounds for the experiments at the standard temperature difference ∆T = 10 K (*n* = 9). Data control with the Grubbs test revealed one set of outliers for the mean biofilm density and the fraction of inorganic compounds for the SST material. This dataset was excluded. More details can be found in the Supplementary Materials Table S2 and S3.

Sensor/Pipe Material	$\mathrm{Mean}\;\mathrm{Biofilm}\;\mathrm{Thickness}\;\bar{L_{F}}\;(µm)$	Mean Biofilm Density(kg/m^3^)	Fraction of Inorganic Compounds (kg/m^3^)
C-PP	276 ± 102 (± 37%)	24 ± 13 (± 54%)	8 ± 5 (± 63%)
SST	170 ± 84 (± 49%)	19 ± 8 (± 42%)	9 ± 4 (± 44%)

**Table 3:** Comparison of the sensor sensitivity for different applied temperature differences of the sensors.

Temperature Difference (∆T)	Mean Biofilm Thickness SST (µm)	Sensitivity SST (µm/a.u.)	Mean Biofilm Thickness C-PP (µm)	Sensitivity C-PP (µm/a.u.)
10 K	170 ± 84 (± 49%)	11	276 ± 102 (± 54%)	50
5 K	121 ± 29 (± 24%)	9	193 ± 58 (± 30%)	52
2 K	161 ± 52 (± 32%)	77	302 ± 59 (± 20%)	100

## Data Availability

The data that support the findings of this study are available on request from the corresponding author.

## Supplementary Materials

The following supporting information can be downloaded at: https://www.mdpi.com/article/10.3390/bios12010018/s1, Table S1. Overview of the different experimental run. Table S2. Results of the Shapiro-Wilk and Kolmogo-rov-Smirnov tests for the parameters mean biofilm thickness, mean biofilm density and fraction of inorganic compounds at the sensor setting ∆T = 10 K for both pipe materials. Table S3. Results of the Shapiro-Wilk and Kolmogorov-Smirnov tests for the parameters mean biofilm thickness, mean biofilm density and fraction of inorganic compounds at the sensor setting ∆T = 5 K for both pipe materials.
