# Supplementary material for: On-Line Monitoring of Biofilm Accumulation on Graphite-Polypropylene Electrode Material Using a Heat Transfer Sensor
Source: Biosensors (Basel). 2021 Dec 30;12(1):18. doi: 10.3390/bios12010018 (PMC8773567; doi:10.3390/bios12010018)
Supplement: Supplementary file 1 [file biosensors-12-00018-s001.zip › biosensors-1482632-supplementary.pdf]

biosensors

MDPI

Supporting Information

On-Line Monitoring of Biofilm Accumulation on Graphite-Polypropylene Electrode Material Using a Heat Transfer Sensor

Andreas Netsch [1,2,*], Harald Horn [1,2] and Michael Wagner [2,3]

[1] DVGW Research Center, Water Chemistry and Water Technology, Engler-Bunte-Ring 9a, 76131 Karlsruhe, Germany; harald.horn@kit.edu
[2] Engler-Bunte Institut, Water Chemistry and Water Technology, Karlsruhe Institute of Technology (KIT), Engler-Bunte-Ring 9a, 76131 Karlsruhe, Germany; michael.wagner@kit.edu
[3] Institute for Biological Interfaces 1 (IBG-1), Institute for Biological Interfaces (IBG), Karlsruhe Institute of Technology (KIT), Hermann-von-Helmholtz-Platz 1, 76344 Eggenstein-Leopoldshafen, Germany
* Correspondence: andreas.netsch@kit.edu

This SI contains 2 pages of additional information, including 3 tables.

Experimental set-up

Table S1. Overview of the different experimental run.

experiment no.	Q (L/min)	Re (-)	ΔT (K)	number of pipes SST/C-PP
A	3.6	3000	10	4/4
B			10	5/5
C1			10	1/1
C2			5	2/2
C3			2	2/2
D1			10	1/1
D2			5	2/2
D3			2	2/2
E			5	5/5

Statistical analysis

Table S2. Results of the Shapiro-Wilk and Kolmogorov-Smirnov tests for the parameters mean biofilm thickness, mean biofilm density and fraction of inorganic compounds at the sensor setting **ΔT=10 K** for both pipe materials.

pipe material	parameter	n	p-value (Shapiro-Wilk)	p-value (Kolmogorov-Smirnov)
C-PP	mean biofilm thickness $\overline{L_F}$	8	0.337	0.560
	mean biofilm density		0.140	0.886
	fraction of inorganic compounds		0.177	0.902

SST	mean biofilm thickness $\overline{L_F}$	9	0.300	0.739
	mean biofilm density		0.004	0.286
	fraction of inorganic compounds		0.003	0.469

In combination with a Grubbs test the statistical analysis revealed one data set of outliers for the mean biofilm density and fraction of inorganic compounds of the SST pipe material. This data set was excluded from the analysis.

Table S3. Results of the Shapiro-Wilk and Kolmogorov-Smirnov tests for the parameters mean biofilm thickness, mean biofilm density and fraction of inorganic compounds at the sensor setting **ΔT=5 K** for both pipe materials.

pipe material	parameter	n	p-value (Shapiro-Wilk)	p-value (Kolmogorov-Smirnov)
C-PP	mean biofilm thickness $\overline{L_F}$	8	0.474	1
	mean biofilm density		0.010	0.213
	fraction of inorganic compounds		0.012	0.303
SST	mean biofilm thickness $\overline{L_F}$	8	0.930	1
	mean biofilm density		0.014	0.607
	fraction of inorganic compounds		0.089	0.800

In addition the Grubbs test did not reveal any outliers for the data sets of either of the pipe materials at the ΔT=5K setting of the sensor.
